# An audit of breast cancer pathology reporting in Australia in 1995

**DOI:** 10.1038/sj.bjc.6690392

**Published:** 1999-05-01

**Authors:** A Kricker, B Armstrong, C Smith, M Bilous, C Camaris, A Mayer, T Psarianos

**Affiliations:** NHMRC National Breast Cancer Centre; and Cancer Control Information Centre, PO Box 572, Kings Cross, NSW 2011, Australia; Tissue Pathology Department, Institute of Clinical Pathology and Medical Research, Westmead Hospital, Westmead NSW 2145, Australia

**Keywords:** breast cancer, audit, pathology reporting, quality measurement, checklist

## Abstract

To measure the quality of pathology reporting of breast cancer and establish a baseline against which future changes can be measured, we audited item completeness in breast cancer reports in Australia in 1995 before the release of specific recommendations from the Australian Cancer Network. Tumour type and size were given in reports of invasive breast cancer for 93% of women, 70% had, in addition, grade and clearance of the margins while only 28% had all recommended information. The most complete items in reports were histological type of breast cancer (99.6% of cases), tumour size (94%, 95% confidence interval (CI) 92–95) and margins of excision (87%, 95% CI 85–89). Histological grade (84%, 95% CI 82–86 of cases) and presence or absence of ductal carcinoma in situ (DCIS) (79%, 95% CI 77–81) were less complete and vessel invasion (61%, 95% CI 58–63) and changes in non-neoplastic breast tissue adjacent to the breast cancer (68%, 95% CI 66–71) the least complete. Less than half the reports of DCIS reported on tumour size (49%, 95% CI 42–57), presence or absence of necrosis (41%, 95% CI 34–49) or nuclear grade (39%, 95% CI 31–46). Around 1500 reports were identified as issued by 147 laboratories and 392 pathologists; 69% of pathologists issued fewer than two reports a month in the audit. We concluded that infrequency of reporting may have contributed to incompleteness of reporting. In addition, we found significant variation across Australian states with some indication that reporting was consistently poor in one state. The audit highlighted areas for improvement for breast cancer reporting in Australia. Research evidence suggests that multifaceted strategies are needed to assist practitioners with implementing more uniform reporting standards. © 1999 Cancer Research Campaign


*British Journal of Cancer* (1999) **80**(3/4), 563–568
© 1999 Cancer Research Campaign
Article no. bjoc.1998.0392

### An audit of breast cancer pathology reporting in Australia in 1995

**A Kricker[1], B Armstrong[2], C Smith[1], M Bilous[3], C Camaris[3], A Mayer[3] and T Psarianos[3]**

[1]NHMRC National Breast Cancer Centre and [2]Cancer Control Information Centre, PO Box 572, Kings Cross, NSW 2011, Australia; [3]Tissue Pathology Department, Institute of Clinical Pathology and Medical Research, Westmead Hospital, Westmead NSW 2145, Australia

**Summary** To measure the quality of pathology reporting of breast cancer and establish a baseline against which future changes can be measured, we audited item completeness in breast cancer reports in Australia in 1995 before the release of specific recommendations from the Australian Cancer Network. Tumour type and size were given in reports of invasive breast cancer for 93% of women, 70% had, in addition, grade and clearance of the margins while only 28% had all recommended information. The most complete items in reports were histological type of breast cancer (99.6% of cases), tumour size (94%, 95% confidence interval (CI) 92–95) and margins of excision (87%, 95% CI 85–89). Histological grade (84%, 95% CI 82–86 of cases) and presence or absence of ductal carcinoma in situ (DCIS) (79%, 95% CI 77–81) were less complete and vessel invasion (61%, 95% CI 58–63) and changes in non-neoplastic breast tissue adjacent to the breast cancer (68%, 95% CI 66–71) the least complete. Less than half the reports of DCIS reported on tumour size (49%, 95% CI 42–57), presence or absence of necrosis (41%, 95% CI 34–49) or nuclear grade (39%, 95% CI 31–46). Around 1500 reports were identified as issued by 147 laboratories and 392 pathologists; 69% of pathologists issued fewer than two reports a month in the audit. We concluded that infrequency of reporting may have contributed to incompleteness of reporting. In addition, we found significant variation across Australian states with some indication that reporting was consistently poor in one state. The audit highlighted areas for improvement for breast cancer reporting in Australia. Research evidence suggests that multifaceted strategies are needed to assist practitioners with implementing more uniform reporting standards.

**Keywords:** breast cancer; audit; pathology reporting; quality measurement; checklist

The pathologist's role in breast cancer management is to make a diagnosis and report key items of information essential to treatment planning. To increase national uniformity in procedures and reporting, the Australian Cancer Network's (ACN) Pathology Working Party released specific recommendations about pathology reporting of breast cancer specimens in 1997 (ACN Pathology Working Party, 1997). These recommendations were that all microscopic reports of invasive breast cancer should contain information on tumour type, size, grade, margins of excision, vessel invasion and changes in adjacent breast tissue. For each case of pure ductal carcinoma in situ (DCIS), recommendations were to report tumour size, nuclear grade, necrosis, architecture and calcification and, in addition, a clear statement about the margins of excision.

The NHMRC National Breast Cancer Center (NBCC) undertook an audit of pathology reporting of breast cancer cases diagnosed in Australia in 1995, before the release of the recommendations, to measure a baseline for the coverage and completeness of reporting by pathologists in Australia of key items in the ACN Working Party's recommendations (ACN Pathology Working Party, 1997).

*Received 17 August 1998*
*Revised 13 November 1998*
*Accepted 23 November 1998*

*Correspondence to:* A Kricker

### MATERIALS AND METHODS

We obtained copies of pathology reports, identified by number only, from Australian state and territory cancer registries for cases of invasive breast cancer or DCIS diagnosed in April and May 1995 (and June in the smallest states and territories). In one State, only three of six laboratories agreed to participate, supplying 57% of the cases expected in that State.

Completeness of reporting of invasive breast cancer was examined for the key items: tumour size, histological type, histological grade, margins of excision, vessel invasion and changes in the adjacent breast tissue. We examined reporting of changes in adjacent breast tissue in two separate items, the presence or absence of DCIS and changes in adjacent non-neoplastic tissue. In addition, we examined the reporting of nodal status of all breast cancers and the presence or absence of an extensive intraductal component (EIC) associated with infiltrating ductal carcinomas nitric oxide synthase (NOS) (no special type).

Reports were classified by the type of diagnostic or therapeutic procedure from which the specimen came. Analyses were based on reports from biopsies and mastectomies which contained information about the breast cancer and excluded fine needle aspirations (FNA), core biopsies and slide reviews.

Completeness was defined as the percentage of reports in which a definite statement about an item, either positive or negative, was made. A data dictionary and coding manual and data collection forms were developed for use by three pathology registrars who were the auditors. Typically, the data form sought to classify each pathology item as present, absent, uncertain whether present or absent or not mentioned. In addition, the grade of a tumour and actual measurements of size or margins were noted when given. One data collection form was completed for each pathology report and there were separate forms for invasive breast cancer and DCIS. The sample size (1600) was chosen to give completeness percentages that had 95% confidence bounds that were not more than 5% on either side of the point estimate.

The extent of agreement among auditors was examined in a sample of 10% of pathology reports that were audited independently by all auditors. The kappa statistic was used to examine variation between pairs of auditors after adjustment for the extent of agreement that would be expected by chance (Cohen, 1960).

Three sets of analyses were undertaken which addressed all reports for each woman, all reports from biopsies and mastectomies and the effects of reporting ‘volume’ on completeness. For each woman with invasive breast cancer, an item was regarded as complete if the biopsy or mastectomy report gave the relevant information. It is possible that the sets of reports available for some women were incomplete sets. Analyses by reporting ‘volume’ were based on the numbers of reports issued by each laboratory or pathologist in April and May 1995.

Ninety-five per cent confidence intervals (CI) were calculated for percentages of complete items using the Normal distribution for numbers of ten or more and exact limits using the F distribution for smaller numbers (Armitage and Berry, 1991). Homogeneity of proportions was tested using the $\chi^2$ distribution and trends in percentages across ordered groups were assessed by the Mantel–Haenszel test for trend (Armitage and Berry, 1991).

### RESULTS

We examined 1563 pathology reports of invasive breast cancer from biopsies and mastectomies for 1409 women diagnosed with breast cancer in all Australian states and territories in 1995. A total of 195 reports of re-excision specimens which showed no residual cancer were excluded. More than half (56%) the women had only one report, 37% had two reports and 7% had three or more.

### Agreement between the auditors

Auditor 1 completed data collection forms for 879 (34%) pathology reports of invasive breast cancer, Auditor 2 completed 1312 (50%) and Auditor 3 completed 399 (15%). Agreement was calculated in 140 pathology reports examined by both Auditors 1 and 2 and 38 reports audited by all three.

The observed agreement between pairs of auditors in classifying completeness was very high for tumour type, size, grade and vessel invasion (89% or more) and lower for clearance of the margins of excision, the presence or absence of DCIS and changes in adjacent non-neoplastic tissue (between 66% and 88%). The values of kappa were slightly lower than the observed percentages of agreement.

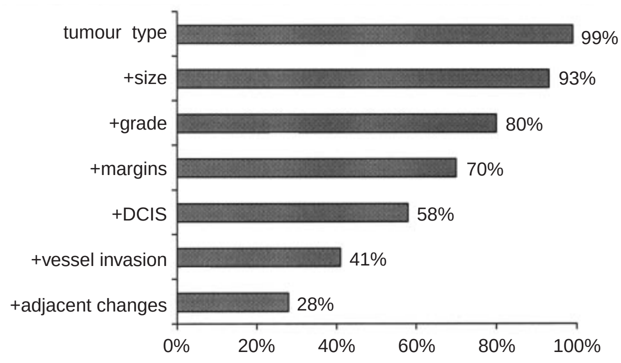

**Figure 1** Percentage of women with invasive breast cancer for whom pathology reports from biopsies and mastectomies gave complete information on key items

### Reports of invasive cancer for each woman

Completeness of reporting invasive cancer for each woman was highest for histological type of cancer (99.6% of cases), tumour size (94%, 95% CI 92–95) and margins of excision (87%, 95% CI 85–89). Histological grade (84%, 95% CI 82–86 of cases) and presence or absence of DCIS (79%, 95% CI 77–81) were reported less while vessel invasion and changes in non-neoplastic breast tissue adjacent to the breast cancer were reported for fewer than 70% of women. Tumour type and size were both stated in 93% of reports and 70% of reports had, in addition, grade and clearance of the margins. Only 28% of all women had all key items reported (Figure 1).

**Table 1** Completeness of key items in 1563 reports of invasive breast cancer from biopsies and mastectomies with variation in rates across Australian states

| Key item | All reports[1] | Completeness of reports by state of residence | | | | | |
|---|---|---|---|---|---|---|---|
| | | State 1 | State 2 | State 3 | State 4 | State 5 | $\chi^2$ (*P*-value) |
| | % (CI) | % (CI) | % (CI) | % (CI) | % (CI) | % (CI) | |
| Tumour size | 89 (87–90) | 87 (84–90) | 93 (91–96) | 86 (82–90) | 91 (86–95) | 87 (76–94) | 14.0 (= 0.02) |
| Tumour type | 99 (99–100) | 100 (99–100) | 100 (99–100) | 99 (99–100) | 97 (93–99) | 100 (95–100) | 17.8 (= 0.003) |
| Histological grade | 80 (78–82) | 80 (77–83) | 85 (82–89) | 74 (69–79) | 85 (79–91) | 74 (63–84) | 18.2 (= 0.003) |
| Margins of excision | 83 (81–85) | 84 (81–87) | 82 (78–86) | 78 (73–83) | 86 (81–92) | 84 (75–93) | 7.5 (= 0.2) |
| Vessel invasion | 57 (55–60) | 54 (50–59) | 56 (51–60) | 54 (47–60) | 72 (65–80) | 60 (49–72) | 17.2 (= 0.004) |
| Presence of DCIS | 75 (73–78) | 75 (72–79) | 74 (70–78) | 72 (66–77) | 80 (74–87) | 84 (75–93) | 8.5 (= 0.1) |
| Changes in adjacent non-neoplastic tissue | 61 (58–63) | 61 (57–65) | 65 (60–69) | 49 (43–55) | 60 (52–68) | 66 (55–77) | 19.9 (= 0.001) |

[1]All reports irrespective of procedure type. Chi-square test for homogeneity across five Australian states (NSW & ACT, Victoria, Queensland, WA, Tasmania) with 4 degrees of freedom. CI, confidence interval; DCIS, ductal carcinoma in situ.

 © Cancer Research Campaign 1999

### Reports of invasive breast cancers from biopsies and mastectomies

Item completeness was examined in greater detail in the 1563 individual reports of invasive breast cancer from biopsies and mastectomies. Of these, 21% gave a definite finding for all key items, 51% had six or seven, and 73% had five or less items complete. As might be expected, less complete information was given in reports which followed a re-excision, especially tumour size (23 percentage points less) and histological grade (22 percentage points less), but also vessel invasion and presence of additional DCIS (10 percentage points less). Reporting of tumour size was high (89%, 95% CI 87–90), and higher (91%, 95% CI 90–93) when there were one or two tumours than when there were three or more (65%, 95% CI 57–73%).

Histological grade was given in the majority (80%, 95% CI 78–82) of reports. Overall, 31% of reports named the Bloom and Richardson grading system, as recommended by the ACN Working Party. Reports with breast cancer size more often gave grade (85% of 1175 reports) than those without (45% of 173). Grade was given less often (33% of 42 reports) for smaller (< 4 mm) than larger (4 mm or more) cancers (85% of 1348 reports).

Given the importance of clear margins for prognosis and treatment decision, reporting was surprisingly variable. Comments about excision margins were made in 83% (95% CI 81–85) of reports but only in 62% (95% CI 59–65) of 1038 reports with clear margins was a measurement of the margin given. Moreover, the auditors indicated that, for 33 of 121 reports (27%, 95% CI 19–35) with a stated margin of 1 mm or less, clearance was, in fact, uncertain.

There was statistically significant variation ($P < 0.05$) by Australian state in completeness of reporting of histological type and grade, size, vessel invasion and changes in adjacent breast tissue (Table 1). Differences in reporting histological type were based on a handful of cases only, while tumour size varied by seven percentage points, histological grade by eleven, changes in adjacent non-neoplastic tissue by 17, and vessel invasion by 18 percentage points. One Australian state had the lowest item completeness in all items (Table 1).

Completeness of reports of tumour size increased significantly ($P < 0.001$) with increasing age of the women, from 85% at < 50 years of age to 93% at 70+ years of age, while there was significantly ($P < 0.001$) less complete reporting of DCIS in women 70+ years (69%) compared with women younger than 70 years (77–78%).

The Pathology Working Party defined an extensive intraductal component (EIC) principally as the presence of ‘a significant amount’ of DCIS in an infiltrating ductal cancer NOS, together with any DCIS in the adjacent breast tissue, noting that there has been confusion about its definition. Few of the 1199 reports of infiltrating ductal carcinomas made comments about EIC status. Thirty-nine per cent described a DCIS component in or adjacent to the invasive breast cancer as ‘less than or greater than 25%’, ‘EIC+ or EIC–’, or ‘extensive, minimal, or moderate’. Six per cent measured the extent of DCIS but did not indicate its EIC status, 20% indicated only that DCIS was present or absent and 35% of reports did not mention DCIS at all.

A statement about lymph nodes was given in 55% (95% CI 52–57) of reports. Most reports mentioning lymph nodes gave the number of nodes examined (97%) and the number positive (98%; 835 of 852 reports).

#### *Different histological types of invasive breast cancers*

Grade was given for 91% (95% CI 89–93) of infiltrating ductal carcinomas NOS compared with only 34% (95% CI 27–41) of lobular carcinomas (Table 2). Tumour size (90%, 95% CI 88–92) and presence or absence of DCIS (82%, 95% CI 80–84) were most often given for infiltrating ductal carcinomas NOS and least often for lobular carcinomas (tumour size, 82%, 95% CI 76–87; statements about DCIS, 35%, 95% CI 28–42).

Grade was given for most (96%, 95% CI 95–98) of the infiltrating ductal carcinomas NOS 3+ mm but only in 35% (95% CI 15–54) of the cancers less than 3 mm. Infiltrating ductal carcinomas NOS with no tumour size had been graded less often (54%, 95% CI 45–63) than reports with size recorded (95%, 95% CI 94–96).

**Table 2** Reporting of key items in reports of invasive breast cancer from biopsies and mastectomies by categories of histological type

| | Histological types | | | |
|---|---|---|---|---|
| Key item | Ductal carcinoma of no special type (*n* = 1199) % (CI) | Ductal carcinoma of special types (*n* = 103) % (CI) | Lobular carcinoma (*n* = 181) % (CI) | Other and unknown type of carcinoma[1] (*n* = 80) % (CI) |
| Tumour size | 90 (88–92) | 96 (92–100) | 82 (76–87) | 83 (74–91) |
| Histological type | 100 (100–100) | 100 (100–100) | 100 (100–100) | 90 (83–97) |
| Histological grade | 91 (89–93) | 50 (41–60) | 34 (27–41) | 60 (49–71) |
| Margins of excision | 84 (82–86) | 83 (75–90) | 79 (73–85) | 76 (67–86) |
| Vessel invasion | 61 (58–64) | 49 (39–58) | 43 (36–50) | 43[2] (32–53) |
| Presence of DCIS | 82 (80–84) | 77 (69–85) | 35 (28–42) | 63[3] (52–73) |
| Changes in adjacent non-neoplastic tissue | 61 (58–64) | 64 (55–73) | 59 (51–66) | 59 (48–70) |

[1]Includes carcinomas of mixed type (63 reports), other type (nine reports) and unknown type (eight reports). [2]A total of 51% (95% CI 38–63) in other carcinoma of mixed type. [3]A total of 71% (95% CI 59–81) in other carcinoma of mixed type.
CI, confidence interval; DCIS, ductal carcinoma in situ.

© Cancer Research Campaign 1999

**Table 3** Completeness of pathology reporting of key items in DCIS reports from biopsies and mastectomies

| Key item | Reports: All n = 170 % (CI) | Biopsies n = 117 % (CI) | Mastectomies n = 53 % (CI) |
|---|---|---|---|
| Tumour size given | 49 (42–57) | 57 (48–66) | 32 (20–45) |
| One or two tumours | 64 (56–71) | 71 (63–79) | 47 (34–61) |
| Three or more or multifocal | 36 (29–44) | 29 (21–37) | 53 (39–66) |
| Margins of excision | 76 (69–82) | 79 (71–86) | 70 (57–82) |
| Nuclear grade given | 39 (31–46) | 43 (34–52) | 30 (18–43) |
| Necrosis given | 41 (34–49) | 42 (33–51) | 40 (26–53) |
| Architecture given | 95 (91–98) | 93 (89–98) | 98 (94–100) |
| Calcification given | 57 (50–64) | 67 (58–75) | 36 (23–49) |

CI, confidence interval.

### *DCIS*

Item completeness for DCIS was measured in 170 reports from biopsies and mastectomies in 145 women (Table 3). Information was most complete (95%, 95% CI 91–98) for architecture: 47% were classified by growth pattern as comedo or solid, cribriform or micropapillary, 33% were said to be mixed type and 15% were no special type. Less complete information was given for excision margins (76%, 95% CI 69–82), tumour size when only one or two tumours were present (64%, 95% CI 56–71) and calcification (57%, 95% CI 50–65). Overall less than half the reports gave tumour size (49%), presence or absence of necrosis (41%) or nuclear grade (39%).

### Completeness of reports of invasive breast cancer by number of reports for pathologists and laboratories

There were 1316 reports in April and May assigned to categories of reporting volume; the pathologist was unknown for an additional 46 cases. Nearly half the pathologists (47%) had issued only one (29%) or two (18%) reports. The majority (69%) of pathologists issued less than five reports of invasive breast cancer during these 2 months.

Only vessel invasion showed some evidence of increasing completeness with increasing volume of reports. Presence or absence of vessel invasion was described more often by pathologists who had issued five or more reports (59–61%) than less than five (44–57%; *P*-value for trend = 0.05) (Table 4).

Of the 129 laboratories identified with 1362 invasive breast cancer reports, most (91%) had issued less than 15 reports in total over the 2-month period while only six had issued 40 or more reports (an average of five a week). The high volume laboratories had more complete information on tumour size (*P* for trend = 0.05), margins of excision and vessel invasion (*P* for trend < 0.05 in each case) (Table 4).

### DISCUSSION

Seventy per cent of women with invasive breast cancer in 1995 in Australia had pathology reports with complete information on

**Table 4** Completeness of key items for invasive breast cancers by categories of pathologists and laboratories

| Key item | Number of reports issued by pathologists: 1–2 (*n* = 244) % (CI) | 3–4 (*n* = 265) % (CI) | 5–6 (*n* = 238) % (CI) | 7–10 (*n* = 341) % (CI) | 10+ (*n* = 228) % (CI) | Trend[1] $\chi^2$ (P-value) |
|---|---|---|---|---|---|---|
| Tumour size | 87 (83–91) | 87 (83–91) | 88 (84–92) | 90 (87–93) | 91 (88–95) | 3.13 (0.08) |
| Tumour type | 100 (98–100) | 99 (97–100) | 99 (97–100) | 99 (97–100) | 100 (98–100) | 0.99 (0.61) |
| Histological grade | 80 (74–85) | 78 (74–83) | 84 (80–89) | 79 (75–83) | 83 (78–88) | 0.62 (0.43) |
| Excision margins | 84 (80–89) | 76 (71–81) | 88 (84–92) | 82 (78–86) | 86 (81–90) | 1.19 (0.28) |
| Vessel invasion | 57 (51–63) | 44 (38–50) | 61 (55–68) | 59 (54–64) | 59 (52–65) | 3.96 (0.05) |
| Presence of DCIS | 77 (72–83) | 72 (67–78) | 76 (71–82) | 78 (73–82) | 70 (64–76) | 0.86 (0.36) |
| Changes in adjacent non-neoplastic tissue | 64 (58–70) | 59 (53–65) | 58 (52–64) | 59 (53–64) | 60 (53–66) | 1.07 (0.30) |

| Key item | Number of reports issued by laboratories: 1–6 (*n* = 247) % (CI) | 7–13 (*n* = 263) % (CI) | 14–25 (*n* = 270) % (CI) | 26–40 (*n* = 260) % (CI) | 40+ (*n* = 320) % (CI) | Trend[1] $\chi^2$ (*P*-value) |
|---|---|---|---|---|---|---|
| Tumour size | 88 (84–92) | 88 (84–92) | 85 (81–89) | 88 (84–92) | 94 (91–96) | 3.95 (0.05) |
| Tumour type | 100 (98–100) | 99 (97–100) | 100 (99–100) | 100 (99–100) | 99 (97–100) | 0.99 (0.61) |
| Histological grade | 82 (77–87) | 78 (73–83) | 78 (73–83) | 78 (73–83) | 86 (82–90) | 1.77 (0.18) |
| Excision margins | 74 (69–80) | 83 (78–87) | 86 (82–90) | 84 (80–89) | 86 (82–90) | 11.22 (0.001) |
| Vessel invasion | 49 (43–55) | 56 (50–62) | 51 (46–57) | 57 (51–63) | 66 (61–71) | 14.45 (0.001) |
| Presence of DCIS | 74 (69–80) | 77 (72–82) | 74 (69–80) | 73 (68–79) | 77 (73–82) | 0.17 (0.68) |
| Changes in adjacent non-neoplastic tissue | 56 (50–62) | 56 (50–62) | 65 (59–71) | 66 (60–72) | 57 (51–62) | 1.04 (0.31) |

[1]The Mantel–Haenszel test for trend with 1 degree of freedom. CI, confidence interval; DCIS, ductal carcinoma in situ.

 © Cancer Research Campaign 1999

each of the four items – histological type, size, grade and clearance of the margins – widely regarded as the key to informed patient management (Nakhleh et al, 1997) and vital for prognosis (Elston and Ellis, 1991; Henson et al, 1991; Wijetunga et al, 1996). Only 28%, however, had reports which gave all the Pathology Working Party's recommended items of information. The deficiencies in individual items such as tumour size (missing for 6% of women), clearance of the margins (missing for 13%) and histological grade (missing for 16%) gave cause for concern.

Compared with earlier Australian audits (Table 5), 1995 pathology reports were much more complete, particularly for histological grade (+15 percentage points), excision margins (+10 percentage points) and DCIS (as much as +36 percentage points). Completeness of reporting of tumour size, in comparison, appeared to have reached a plateau, with little change (+1 percentage point) from 1992 after a larger improvement (+10 percentage points) from 1989 (Table 5).

Reporting of DCIS was relatively poor. Although most reports gave architechture and three-quarters stated whether margins were involved or clear, less than half gave size, nuclear grade or presence or absence of necrosis, suggesting a lack of a uniform approach to histological reporting of these tumours. A 1995 survey of specialist clinicians, mainly in the USA but including a handful in Australia, ranked size and margin status (99–100% of surgeons and oncologists), tumour architecture (93–98%) and nuclear grade (88–94%) as the most desired information for clinical management of DCIS (Nakhleh et al, 1997).

The ACN's Pathology Working Party recommended that all invasive carcinomas have 'the Elston modification of Bloom and Richardson's grading … applied' (ACN Pathology Working Party, 1997). The differences in completeness of reporting grade between ductal (91% graded), lobular (34%) and other carcinomas (55%) suggests that the grading of other than ductal carcinomas may be a relatively new approach in Australian laboratories. Relative newness may also account for the incompleteness of DCIS reporting when it is present alone and as a component of infiltrating ductal carcinomas NOS. Reports with breast cancer size more often stated grade than those without. This coincidence may suggest that when standard procedures include assessment of one of these features, routine assessment of the other is also likely.

Pathology reports of invasive breast cancers in 1995 in Australia had higher levels of information of size (complete for 94% of women) and histological grade (84%) than a UK health region in 1992 (size and grade each 74% complete; Ma et al, 1997). The UK study, on the other hand, had more complete information (94%) on surgical margins of excision than in Australia (84%; Ma et al, 1997).

We found some evidence that completeness of reporting varied with reporting volume particularly for laboratories rather than individual pathologists. Reporting of cancer size, margins of excision and vessel invasion all increased significantly with laboratory reporting volume. A similar trend was found in the 1992 audit in NSW in which reporting was more complete from pathologists who issued at least 2–3 reports a month or were based in teaching hospitals (Bilous et al, 1995). Volume of institutional reporting in the UK was also associated with item completeness, although inconsistently since reporting of tumour size fell as volume increased while margins of excision and histological grade were more completely reported as volume increased (Ma et al, 1997).

Many pathologists, in assessing breast cancers, would have carried out appropriate procedures (Rippey, 1996; Bull et al, 1997). Failure to state key information in pathology reports has been attributed to inadvertant omission (Bull et al, 1997), the tendency to substitute telephone consultation for completeness in reporting (Miller and Slater, 1996; Hammond and Flinner, 1997), perceptions of clinicians' information needs (Nakhleh et al, 1997) and significant uncertainty about best practice because of changing knowledge of the importance of items for choices about management and prognosis (Bull et al, 1997).

There was important variation among Australian states in completeness of reporting of margins of excision, vessel invasion and changes in adjacent tissue. Although margins of excision and

**Table 5** Completeness of pathology reporting in studies in Australia and the UK

| Key Item | Complete items in pathology reports for breast cancer cases | | | | |
|---|---|---|---|---|---|
| | Australia 1995 % | UK 1992[1] % | NSW 1992[2] % | NSW 1986–1994[3] % | WA 1989[4] % |
| Tumour type | | | 94 | 99 | 97 |
| Tumour size | 94 | 74 | 93 | 46 | 84 |
| Margins of excision | 87 | 94 | 77 | | 60 |
| Histological grade | 84 | 84 | 69 | 47 | 45 |
| Presence of DCIS | 79 | | | | 43 |
| Adjacent non-neoplastic tissue | 68 | | | | |
| Changes in non-involved breast tissue (NSW) | | | 60 | | |
| Vessel invasion | 61 | | | | |
| Lymphatic invasion | | | 34 | 21 | |
| Vascular invasion | | | 24 | 9 | 27 |
| Axillary surgery mentioned | | | 97 | | 87 |
| number of nodes involved | | 88 | 100 | 98 | 99 |

[1]Ma M, Bell J, Campbell S et al (1997) Breast cancer management: is volume related to quality? *Br J Cancer* **75**: 1652–1659. [2]Bilous M, McCredie M, Porter L (1995) Adequacy of histopathology reports for breast cancer in NSW. *Pathology* **27**: 306–311. [3]Wijetunga LHR, Carmalt HL, Gillett DJ (1996) A review of pathology reporting for breast cancer. *Aust NZ J Surg* **66**: 723–726. [4]Harvey JM, Sterrett GF, Parsons RW et al (1995) Breast cancer in Western Australia in 1989: IV. Summary of histopathological assessment in 655 cases. *Pathology* **27**: 12–17.

© Cancer Research Campaign 1999

vessel invasion were increasingly completely reported as volume of reporting in laboratories increased, three States had consistently low levels of information. A strong State-level influence was suggested by the observation that one State had the lowest levels of reporting on all items other than tumour type (Table 1). We do not know the reason for this difference between States.

The change from more to less radical surgical procedures has meant that pathology information is not only important in giving a prognosis and deciding on post-operative adjuvant therapy for the woman with breast cancer, but is also important for monitoring the quality of health care. Measurement of quality in surgery, by auditing surgical margins and the numbers of nodes excised, and the performance of early detection programmes by monitoring size, grade and nodal status rely on pathology information. Given the higher incidence of DCIS since the introduction of mammographic screening, it is important also that reporting of the pathology of these cancers is more uniform and of high quality.

Use of a standard form or checklist is the one practice found to be significantly associated with an increased completeness of reporting of selected items in pathology reports of cancer (Zarbo, 1992; Bilous et al, 1995; Wijetunga et al, 1996; Rippey, 1996; Shepherd and Quirke, 1997; Bull et al 1997). Synoptic reporting was recommended by the ACN's Pathology Working Party and its widespread adoption would be expected to lead to further increases in completeness of reporting. These increases would, in turn, be expected to lead to improved outcomes and quality of care. One option to increase the use of synoptic reporting in Australia might be to consider its adoption as an accreditation requirement for pathologists and laboratories.

By measuring completeness of item information, we have undertaken the first steps in ascertaining the quality of pathology reporting. We believe that this simple process has highlighted important gaps in reporting breast cancers in Australia in 1995. We have circulated a summary of audit outcomes to all members of the Royal College of Pathologists Australia and the complete report to major health organizations. Passive, dissemination-only strategies, however, are known to be generally ineffective in improving practice (Bero et al, 1998). While more intensive, 'multifaceted' strategies that combine methods, such as locally-based consensus processes and participation in audit with feedback, would probably be more effective with pathologists and laboratories (Bero et al, 1998), our audit was designed to assess performance at a national or state, and not an individual, level. A much larger sample size would be needed to obtain sufficiently precise estimates of individual performance that could be supplied as individual feedback.

The existence of the ACN's recommendations speaks to a local consensus on the value of key items in breast cancer care. What is needed is a mechanism to assist laboratories and individuals to implement more uniform reporting standards (Bull et al, 1997; Shepherd and Quirke, 1997; Nakhleh et al, 1997). Research evidence suggests that consistently effective interventions for change in health professionals are multifaceted, flexible, systematized, focussed on everyday practice and locally based, and include self-assessment and feedback, practice reminders, consensus processes and interactive educational meetings (du Boulay, 1997; Bero et al, 1998). Currently, no one organization has a mandate to ensure that research findings about pathology reporting standards and interventions to improve professional performance are translated into improvements in everyday practice.

### ACKNOWLEDGEMENTS

We thank the pathologists who participated by agreeing to their reports being included in the audit and the Australian State and Territory Cancer Registries for supplying the pathology reports.

### REFERENCES

ACN Pathology Working Party (1997) *The Pathology Reporting of Breast Cancer. A Guide for Pathologists, Surgeons and Radiologists*. Sydney: Australian Cancer Network

Armitage P and Berry G (1991) *Statistical Methods in Medical Research*. Blackwell Scientific: Oxford

Bero LA, Grilli R, Grimshaw JM, Harvey E, Oxman AD and Thomson MA (1998) Closing the gap between research and practice: an overview of systematic reviews of interventions to promote the implementation of research findings. The Cochrane Effective Practice and Organization of Care Review Group. *Br Med J* **317**: 465–468

Bilous M, McCredie M and Porter L (1995) Adequacy of histopathology reports for breast cancer in New South Wales. *Pathology* **27**: 306–311

Bull AD, Biffin AH, Mella J, Radcliffe AG, Stamatakis JD, Steele RJ and Williams GT (1997) Colorectal cancer pathology reporting: a regional audit. *J Clin Pathol* **50**: 138–142

Cohen J (1960) A coefficient of agreement for nominal scales. *Educational and Psychological Measurement* **20**: 37–46

du Boulay C (1997) Continuing medical education for pathologists: an evaluation of the Royal College of Pathologists' Wessex pilot scheme. *J Clin Pathol* **50**: 1022–1026

Elston CW and Ellis IO (1991) Pathological prognostic factors in breast cancer. I. The value of histological grade in breast cancer: experience from a large study with long-term follow-up. *Histopathology* **19**: 403–410

Hammond EH and Flinner RL (1997) Clinically relevant breast cancer reporting: using process measures to improve anatomic pathology reporting. *Arch Pathol Lab Med* **121**: 1171–1175

Harvey JM, Sterrett GF, Parsons RW, Fitzgerald CJ, Jamrozik K, Dewar JM, Byrne MJ, Ingram DM, Sheiner HM (1995) Breast cancer in Western Australia in 1989: IV. Summary of histopathological assessment in 655 cases. *Pathology* **27**: 12–17

Henson DE, Ries L, Freedman LS and Carriaga M (1991) Relationship among outcome, stage of disease, and histologic grade for 22,616 cases of breast cancer. The basis for a prognostic index. *Cancer* **68**: 2142–2149

Ma M, Bell J, Campbell S, Basnett I, Pollock A and Taylor I (1997) Breast cancer management: is volume related to quality? Clinical Advisory Panel. *Br J Cancer* **75**: 1652–1659

Miller JM and Slater DN (1996) Do histopathology reports of primary cutaneous melanoma contain enough essential information? *J Clin Pathol* **49**: 202–204

Nakhleh RE, Jones B and Zarbo RJ (1997) Mammographically directed breast biopsies: a College of American Pathologists Q-Probes study of clinical physician expectations and of specimen handling and reporting characteristics in 434 institutions. *Arch Pathol Lab Med* **121**: 11–18

Rippey JJ (1996) Standardisation of histopathology reports. *J Clin Pathol* **49**: 864–865

Shepherd NA and Quirke P (1997) Colorectal cancer reporting: are we failing the patient? *J Clin Pathol* **50**: 266–267

Wijetunga LH, Carmalt HL and Gillett DJ (1996) A review of pathology reporting for breast cancer. *Aust NZ J Surg* **66**: 723–726

Zarbo RJ (1992) Interinstitutional assessment of colorectal carcinoma surgical pathology report adequacy. A College of American Pathologists Q-Probes study of practice patterns from 532 laboratories and 15 940 reports. *Arch Pathol Lab Med* **116**: 1113–1119

*© Cancer Research Campaign 1999*
